# The Use of Hypnotics and Mortality - A Population-Based Retrospective Cohort Study

**DOI:** 10.1371/journal.pone.0145271

**Published:** 2015-12-28

**Authors:** Tzuo-Yun Lan, Ya-Fang Zeng, Gau-Jun Tang, Hui-Chuan Kao, Hsien-Jane Chiu, Tsuo-Hung Lan, Hsiao-Feng Ho

**Affiliations:** 1 Institute of Hospital & Health Care Administration, National Yang-Ming University, Taipei, Taiwan; 2 Department of Public Health, Tzu Chi University, Hualien, Taiwan; 3 Min-Sheng General Hospital, Taoyuan, Taiwan; 4 Department of Psychiatry, Taichung Veterans General Hospital, Taichung, Taiwan; 5 National Health Insurance Administration, Ministry of Health and Welfare, Taipei, Taiwan; Kagoshima University Graduate School of Medical and Dental Sciences, JAPAN

## Abstract

**Background:**

Sleep disorders, especially chronic insomnia, have become major health problem worldwide and, as a result, the use of hypnotics is steadily increasing. However, few studies with a large sample size and long-term observation have been conducted to investigate the relationship between specific hypnotics and mortality.

**Methods:**

We conducted this retrospective cohort study using data from the National Health Insurance Research Database in Taiwan. Information from claims data including basic characteristics, the use of hypnotics, and survival from 2000 to 2009 for 1,320,322 individuals were included. The use of hypnotics was divided into groups using the defined daily dose and the cumulative length of use. Hazard ratios (HRs) were calculated from a Cox proportional hazards model, with two different matching techniques to examine the associations.

**Results:**

Compared to the non-users, both users of benzodiazepines (HR = 1.81; 95% confidence interval [CI] = 1.78–1.85) and mixed users (HR = 1.44; 95% CI = 1.42–1.47) had a higher risk of death, whereas the users of other non-benzodiazepines users showed no differences. Zolpidem users (HR = 0.73; 95% CI = 0.71–0.75) exhibited a lower risk of mortality in the adjusted models. This pattern remained similar in both matching techniques. Secondary analysis indicated that zolpidem users had a reduced risk of major cause-specific mortality except cancer, and that this protective effect was dose-responsive, with those using for more than 1 year having the lowest risk.

**Conclusions:**

The effects of different types of hypnotics on mortality were diverse in this large cohort with long-term follow-up based on representative claims data in Taiwan. The use of zolpidem was associated with a reduced risk of mortality.

## Introduction

Sleep disorders are a universal public health problem. In the United States, approximately 50 to 70 million people suffer from sleep disorders [1], and most of these cases can progress to chronic insomnia. The prevalence of chronic insomnia is rising, with affected patients accounting for 10% of the total population [1]. Chronic insomnia has been associated with adverse health outcomes and poor quality of life [2].

Sleep disorders can be treated using medications and psychological therapy, of which hypnotics are the most common treatment [3]. Hypnotics include traditional benzodiazepines (BZDs) and the new generation of non-BZDs. Approximately 10 to 15% of the population in the United States and Europe [4] and 3.5 to 5.4% of the population in Japan [5] are treated with hypnotics.

**Fig 1:**
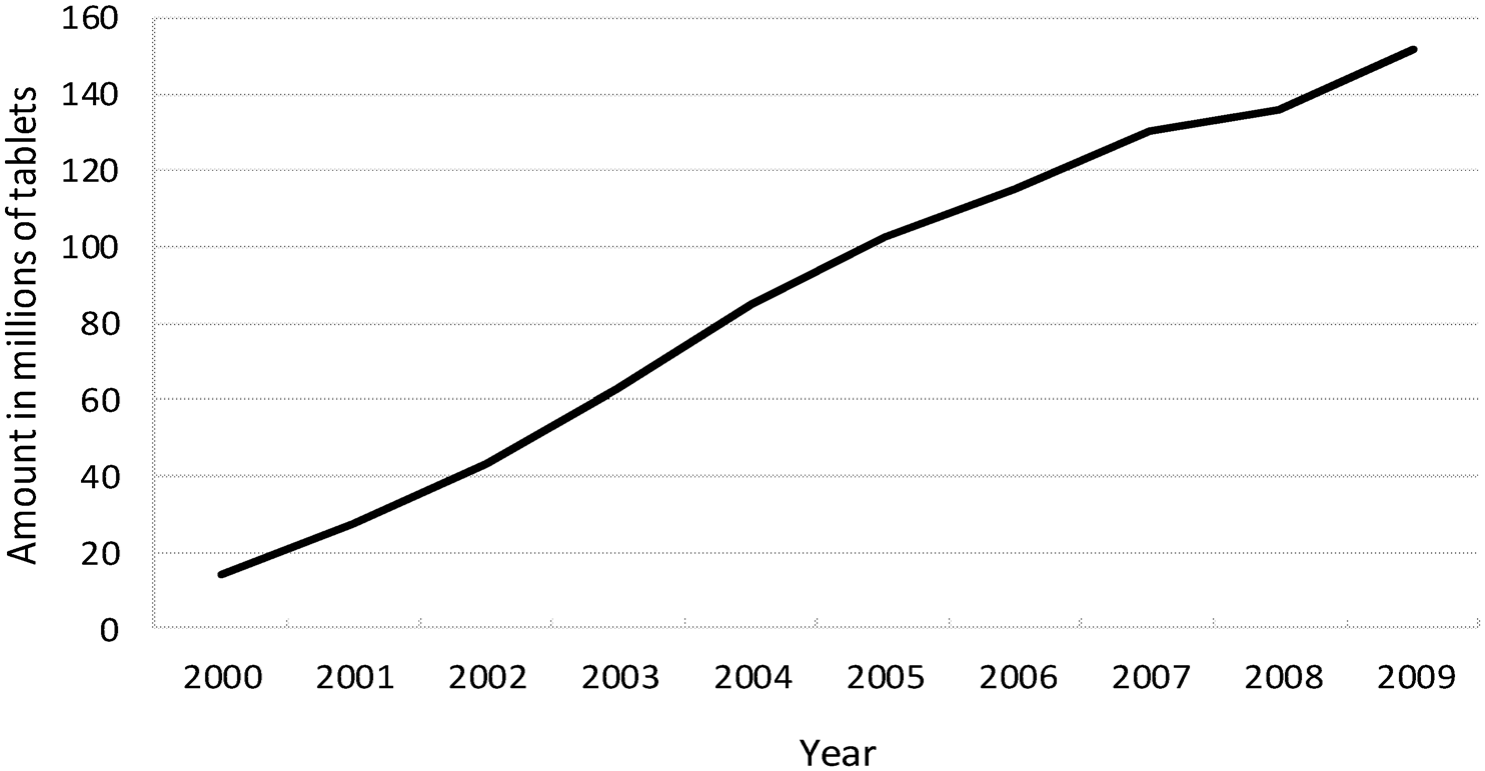
Estimated zolpidem usage from 2000 to 2009 in Taiwan. (Estimates were calculated by dividing the annual cost by the average price for a zolpidem tablet. Source: Bureau of National Health Insurance in Taiwan).

The prevalence of sleep disorders in Taiwan is high and continues to increase. According to the results of a recent survey, the prevalence of sleep disorders in Taiwan is 21.8%, which indicates that approximately 4.8 million people have sleep disorders, of whom 2 million suffer from chronic insomnia [6]. The use of hypnotics, especially the new generation non-BZD drug zolpidem, is relatively high. Fig 1 shows the recent trend of zolpidem usage in Taiwan [7].

Because chronic insomnia and the use of hypnotics are common, it is important to investigate the health effects of the long-term usage of these medications. However, due to restrictions regarding follow-up time and research subjects, it is difficult to undertake phase 4 clinical trials. Consequently, several population-based studies have been conducted to examine the relationship between the use of hypnotics and mortality [8–20], but no consistent results have been established. Furthermore, most of the aforementioned studies conducted surveys on hypnotics as a whole rather than on specific drugs, and the few studies that focused on specific hypnotics did not include a sufficient number of cases or a relatively long period of observation, such as more than five years. Therefore, to better understand the long-term effects of different types of hypnotics on mortality, we performed this study using data from a large-scale health insurance claims database that contained complete records of medical visits, including medications, over a period of 10 years.

## Methods

### Data source

In 1995, the National Health Insurance (NHI) program was initiated in Taiwan, and the coverage rate now exceeds 97% [21]. The research-oriented NHI Research Database (NHIRD) was established using claims data from the NHI program. One million people were randomly sampled from the NHIRD in 2000, 2005, and 2010. For each cohort from the three different years, the annual claims data of the enrollees were merged. This database is therefore representative and suitable for use in the long-term medical follow-up studies [22]. This study was reviewed and approved by the Institutional Review Board of Taipei Veterans General Hospital (VGHIRB No.: 2012-10-006BCY). The data were analyzed anonymously, and informed consent was not required from the study sample.

### Study sample

**Fig 2:**
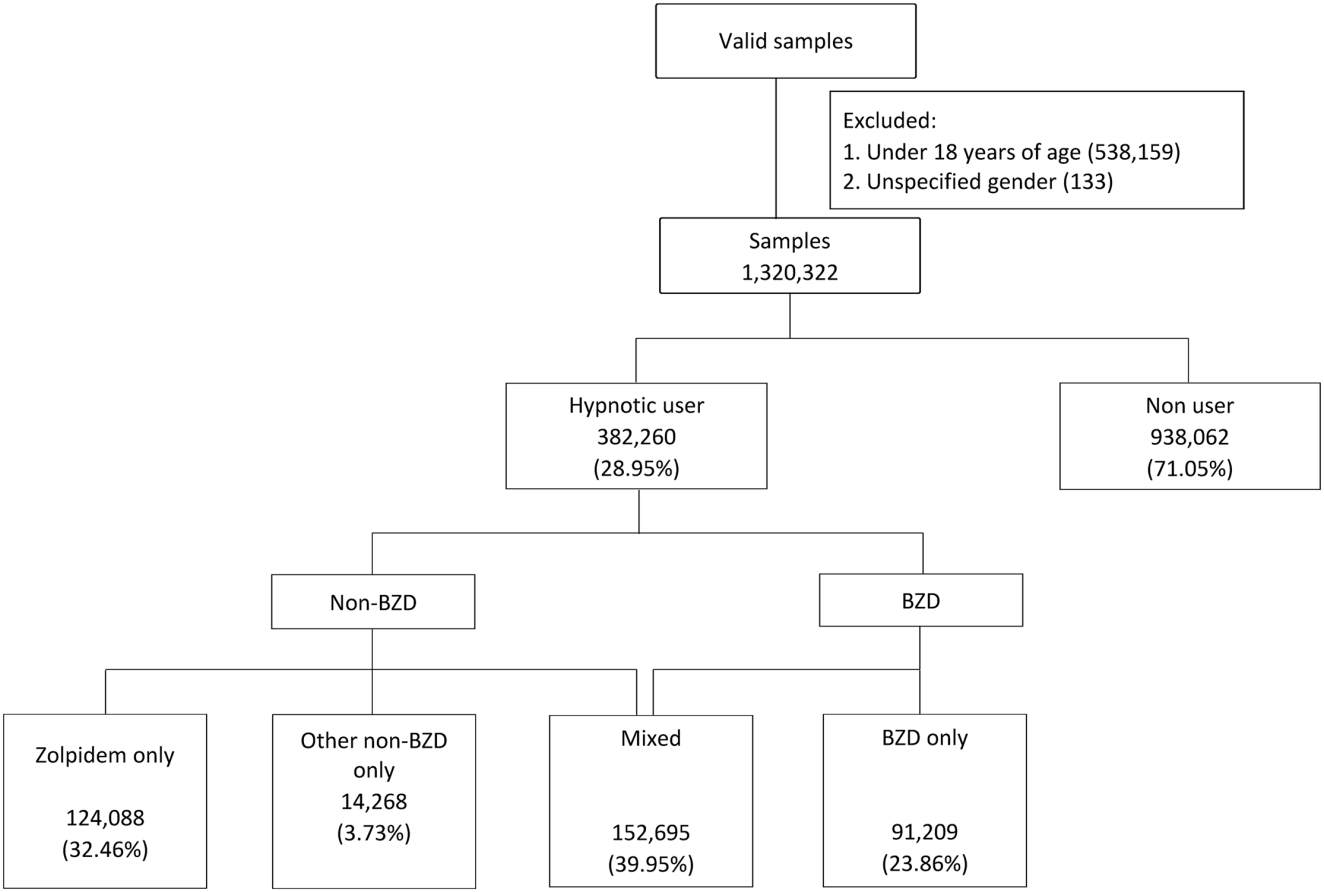
Flowchart of sample selection and classification. (“Mixed” refers to the use of a combination of zolpidem and other non-BZDs or non-BZDs and BZDs).

We used two cohorts, one for 2000 and the other for 2005, from the NHIRD. The cumulative information from 2000 to 2009 regarding all selected enrollees’ medical utilization was collected. The original sample comprised of 1,858,614 people. After eliminating 538,159 people who were under 18 years of age, and 133 people with unspecified gender, the sample used for analysis contained 1,320,322 people. A detailed description of the sample selection process is illustrated in Fig 2.

### Medication usage and other relevant information

Hypnotics were divided into BZDs and non-BZDs. The generic BZDs drugs marketed in Taiwan include the BZDs brotizolam, estazolam, flunitrazepam, flurazepam, lormetazepam, midazolam, nitrazepam, and triazolam, and the non-BZDs include zaleplon, zolpidem, and zopiclone. Among our study subjects, 29% had taken hypnotics, of whom 70% had been prescribed with zolpidem. In light of the widespread use of zolpidem in Taiwan, this study investigated the relationship of mortality with the usage of zolpidem and other hypnotics. Consequently, the study subjects were categorized into five groups: those who took only zolpidem, those who took only non-BZDs apart from zolpidem, those who took only BZDs, those who took medications from more than one of the preceding three categories (combining zolpidem with another non-BZD, zolpidem with a BZD, or a non-BZD with a BZD), and those who did not take any hypnotics as a comparison group. (Fig 2). The NHIRD includes information on medicine dosages, and thus it was also possible to analyze the dose-response relationship between hypnotics and death. Considering the comparability between different drugs, we used the defined daily dose (DDD) [23], which standardizes the amount of drug consumption; we also calculated the cumulative defined daily dose (cDDD) for each subject during the study period. In addition, the dose in each hypnotic group was divided into three categories (less than 7 cDDD, 7–30 cDDD, and more than 30 cDDD).

Age and gender for the subjects were included in our research data. The medical behavior of hypnotics users may be different from that of the general population (non-users). For example, most of the hypnotic drugs in this study are prescribed directly through clinics and thus the distribution of the main medical facility of hypnotics users may differ from that of the general population. Therefore, data on level of main medical facility was included in the analysis. The level and classification of main medical facility that the study subjects most commonly visited comprised of medical centers, regional hospitals, local hospitals, and clinics. Other factors associated with the use of hypnotics included comorbidities and area of residence. Comorbidities were assessed using the widely adopted Charlson comorbidity index (CCI) [24], which includes 17 major diseases including cardiovascular conditions, chronic pulmonary disease, cancer, kidney diseases, diabetes, digestive diseases, and AIDS. However, because the CCI does not include psychological diseases or mental disorders, we also used the Elixhauser comorbidities [25], which cover a total of 30 diseases including alcoholism, drug abuse, depressive disorders, and mental disorders. The Elixhauser comorbidities represent the number of diseases an individual has. Diseases were verified using the subjects’ primary and secondary diagnosis codes as recorded in the NHI claims data from 2000 to 2009 (according to the International Classification of Diseases, 9th Revision, Clinical Modification [ICD-9-CM]). A particular disease was verified if a certain code appeared in the data more than three times. The CCI used in this study referenced the Deyo edition [26], and the original Elixhauser comorbidities measure was adopted. Area of residence was divided into northern, central, southern, and eastern areas corresponding to the administrative regions in Taiwan.

### Follow-up time and mortality outcome

The observation of study subjects began on January 1, 2000, and ended on the date of death for those who died or on December 31, 2009, for those who survived. The date of death was determined by examining the enrollment status according to the NHRD, and was verified using the medical claims data for the last record before death. From 2000 to 2009, 104,699 people died. The mean duration of follow-up was 9.66 years (standard deviation = 1.40). In addition to overall causes of death, we also analyzed the specific causes of death in the zolpidem group. The causes of death were classified based on the primary diagnosis related to mortality in the claims data of those who died during the study period into six main groups based on the number of deaths as follow: cancer (ICD-9-CM codes 140–208), respiratory conditions (ICD-9-CM codes 460–519), cardiovascular conditions (ICD-9-CM codes 390–459), digestive diseases (ICD-9-CM codes 520–579), accidents (ICD-9-CM codes 800–949), and others.

### Statistical analysis

The characteristics of study samples including the level of main medical facility, area of residence, and CCI or Elixhauser comorbidities were updated from the baseline throughout the study period. Differences in these characteristics among the different hypnotics and comparison groups were tested using the chi-square and Student’s t tests. Subsequently, the Cox proportional hazard model was used to analyze the relations among different hypnotics groups and the risk of mortality with or without adjusting for other variables. To better examine the effect of variables considered to be potential confounders related to the use of hypnotics and mortality, age, gender, and other covariates including the level of main medical facility, area of residence, cDDD, and CCI or Elixhauser comorbidities were added into the model using the forward stepwise method. Standard techniques were used to test model validity including interaction and collinearity. Different models yielded similar results and the model without interaction or stratification was presented. In addition, the influence of cDDD on mortality was also analyzed. The subjects who died within 1 year from the beginning of the observation were considered to have been potentially influenced by undiagnosed diseases, and we therefore determined whether omitting these subjects influenced or changed the results of the analysis.

To verify whether the results were robust and to minimize the potential effect of treatment selection bias, subjects were matched by age and gender and separately by propensity score matching (PSM) to calculate both the unadjusted and adjusted risks of mortality. Age in years and gender were individually matched between those who used hypnotics and those who did not. The matched pair ratio for both groups was 1:1. The PSM score was the predicted value produced in the logistic regression model in which the use of hypnotics was the dependent variable and the other measures included in the study were independent variables. The caliper matching method based on the score was then applied with a 1 to 1 match between the two groups. Comparisons of basic characteristics between groups before matching, after age and gender matching, and after PSM matching suggested that comparability for both groups after matching increased (data not shown). The same procedures were conducted for the age- and gender-matched sample and the PSM sample.

The secondary analysis was restricted to the specific causes of death for the subjects who took only zolpidem. Because the cDDD value was a cumulative amount affected by daily dosage and total duration of use, both variables were considered for the zolpidem users. Regarding dosage, in Taiwan, there are two available single doses of zolpidem: 6.5 mg and 10 mg. The subjects in this research mainly took 10 mg per day, with only a few subjects taking 6.5 mg or more than 10 mg (66 subjects took 6.5 mg [0.01%], 704,810 subjects were administered 10 mg [99.06%], and 6,616 subjects took more than 10 mg [0.93%]). Therefore, only the subjects taking a daily dosage of 10 mg zolpidem (124,086 people) were analyzed. To further examine how the duration of use influenced the risk of mortality, the total number of days in which the medicine was taken during the study period was divided into four groups: less than 30 days, 30–179 days, 180–364 days, and more than 365 days. We used SAS version 9.3 and SPSS version 20 for all statistical analyses. The level of significance was set at 0.05. There were no conflicts of interest in this study.

## Results

### Basic characteristics of the study subjects

Table 1 shows the descriptive statistics of the characteristics of the study sample, which include the distribution of age, gender, comorbidity, levels of primary medical institutions, and area of residence for various groups of hypnotics use. Compared to the non-users, the users comprised slightly more women, older people, and people having more diseases. There were also differences in the area of residence and primary medical institutions, indicating that the treatment of hypnotics was influenced by medical levels and areas. Excluding the subjects who took a combination of two hypnotics or more, zolpidem was the most widely used prescription hypnotic, followed by BZDs.

**Table 1:** Basic characteristics of the different hypnotic groups in the study sample (N = 1,320,322).

	Hypnotic user				Non-user
	Non-BZDs		BZDs only	Mixed	
	Zolpidem only	Other non-BZDs only			
N(%)	124,088(9.40)	14,268(1.08)	91,209(6.91)	152,695(11.56)	938,062(71.05)
Age (mean ± SD)	46.69±15.98	49.31±17.55	50.00±17.60	52.79±16.88	40.39±14.84
Gender					
Male (%)	49,709(40.06)	6,644(46.57)	45,499(49.88)	65,252(42.73)	491,271(52.37)
Female (%)	74,379(59.94)	7,624(53.43)	45,710(50.12)	87,443(57.27)	446,791(47.63)
CCI	1.45±1.83	1.58±1.88	1.76±2.07	2.53±2.38	0.66±1.23
Elixhauser	1.63±1.61	1.69±1.65	1.82±1.72	2.91±2.04	0.73±1.15
Level of main medical facility					
Clinic	49,345(39.77)	2,947(20.65)	25,130(27.55)	39,130(25.63)	694,327(74.02)
Local hospital	24,033(19.37)	3,600(25.23)	20,440(22.41)	36,103(23.64)	108,047(11.52)
Regional hospital	27,338(22.03)	4,796(33.61)	25,835(28.33)	44,246(28.98)	76,077(8.11)
Medical center	23,372(18.84)	2,925(20.50)	19,804(21.71)	33,216(21.75)	59,611(6.35)
Area of residence					
North	56,043(45.16)	5,766(40.41)	36,142(39.63)	63,619(41.66)	427,042(45.52)
Central	29,895(24.09)	5,094(35.70)	24,166(26.50)	42,521(27.85)	210,375(22.43)
South	33,044(26.63)	2,496(17.49)	25,618(28.09)	37,404(24.50)	267,269(28.49)
East	5,106(4.11)	912(6.39)	5,283(5.79)	9,151(5.99)	33,376(3.56)

Chi-square tests or Student’s *t* tests were all significant when compared with results for non-users.

BZDs = benzodiazepines; SD = standard deviation; CCI = Charlson comorbidity index.

Mixed indicates those who used at least two types of hypnotics: zolpidem and other non-BZDs, or non-BZDs and BZDs.

### The use of hypnotics and the risk of mortality

The crude risks of mortality for each of the studied variable are presented in Table 2. All of the subjects who took hypnotics had a higher crude risk of death than those who did not. The highest crude risk of mortality was attributed to the use of BZDs, followed by the mixed use of hypnotics, non-BZDs, and zolpidem. In addition, gender, age, comorbidities, medical facilities, and area of residence were individually associated with death, suggesting that these covariates were potential confounding factors. Male gender, older age, and higher number of comorbidities were associated with a higher risk of death. An increasing trend between medical facility and death was observed, with more complicated cases in medical centers having the highest risk. People in different areas of residence also had different mortality risks.

**Table 2:** Crude risk of mortality by hypnotics groups and other basic characteristics (N = 1,320,322).

Characteristic	N (%)	Person-year	Number of deaths (%)	HR^a^ (95% CI)
Hypnotic				
Non-user	938,062 (71.05)	9,123,819	51,934 (5.54)	1.00
Zolpidem only	124,088 (9.40)	1,214,731	7,878 (6.35)	1.14(1.11–1.17)
Other non-BZDs only	14,268 (1.08)	135,420	1,560 (10.93)	2.03(1.93–2.14)
BZDs only	91,2090 (6.91)	837,515	17,176 (18.83)	3.65(3.59–3.71)
Mixed	152,695 (11.56)	1,439,246	26,151 (17.13)	3.23(3.18–3.27)
Gender				
Male	658,375 (49.86)	6,310,565	62,911 (9.56)	1.00
Female	661,947 (50.14)	6,440,166	41,788 (6.31)	0.65(0.64–0.66)
Age	1,320,322 (100.00)	12,750,731	104,699 (100.00)	1.06(1.06–1.06)
CCI	1,320,322 (100.00)	12,750,731	104,699 (100.00)	1.40(1.40–1.40)
Elixhauser	1,320,322 (100.00)	12,750,731	104,699 (100.00)	1.33(1.33–1.33)
Level of main medical facility				
Clinic	810,879 (61.42)	7,933,733	39,200 (4.83)	1.00
Local hospital	138,928 (10.52)	1,822,959	19,979 (14.38)	2.55(2.51–2.60)
Regional hospital	178,292 (13.50)	1,688,498	22,645 (12.70)	2.73(2.69–2.78)
Medical center	192,223 (14.56)	1,305,541	22,875 (11.90)	3.12(3.07–3.18)
Area of residence				
North	588,612 (44.58)	5,693,987	44,013 (7.48)	1.00
Central	312,051 (23.63)	3,010,296	25,284 (8.10)	1.09(1.07–1.10)
South	365,831 (27.71)	3,530,234	30,236 (8.27)	1.11(1.09–1.13)
East	53,828 (4.08)	516,214	5,166 (9.60)	1.30(1.26–1.34)

Mixed indicates those who used at least two types of hypnotics: zolpidem and other non-BZDs, or non-BZDs and BZDs.

BZDs = benzodiazepines; CCI = Charlson comorbidity index; HR = hazard ratio; CI = confidence interval.

Standard errors of age, CCI, and Elixhauser were extremely small; consequently the 95% CI of these variables was approximately equal to the hazard ratio.

^a^ HR has not been adjusted for other covariates.

Table 3 shows the risk of mortality for the subjects taking hypnotics following adjustments for gender, age, comorbidities, primary medical institutions, and area of residence. Compared to the group that did not take hypnotics, the BZD and mixed hypnotics groups had the highest risks of mortality, whereas those who took other non-BZDs had a borderline lower risk or no statistically significant risk of mortality. Finally, the subjects who took only zolpidem had the lowest risk of mortality, with an adjusted risk of mortality 27–36% lower than for those who did not take hypnotics. Moreover, in the cDDD model, the mixed hypnotics and BZD groups exhibited higher risks of mortality, the zolpidem group showed lower risks of mortality, and no significant difference was found in the other non-BZD group. The model eliminating subjects who were died within 1 year after the beginning of the study showed similar results to those in the original model. In this model, the zolpidem group had an approximately 21–29% lower risk of mortality, and this remained significantly low compared to the group that did not take hypnotics.

**Table 3:** Adjusted risk of mortality for the hypnotic groups.

	Full sample model (N = 1,320,322)				1-year lag model (N = 1,313,147)			
	N (%)	HR^a^(95% CI)	HR^b^ (95% CI)	HR^c^ (95% CI)	N (%)	HR^a^(95% CI)	HR^b^ (95% CI)	HR^c^ (95% CI)
Non-user	938,062(71.05)	1.00	1.00	1.00	932,602(71.02)	1.00	1.00	1.00
Zolpidem only	124,088*(9.40)	0.85(0.83–0.87)	0.64(0.63–0.66)	0.73(0.71–0.75)	123,984*(9.44)	0.94(0.92–0.96)	0.71(0.69–0.73)	0.79(0.78–0.81)
<7 (cDDD)	30,270*(2.29)	0.98(0.94–1.02)	0.76(0.73–0.80)	0.85(0.81–0.88)	30,241*(2.30)	1.08(1.03–1.13)	0.84(0.80–0.88)	0.93(0.89–0.97)
7–30	49,919*(3.78)	0.80(0.77–0.83)	0.62(0.60–0.64)	0.68(0.66–0.71)	49,879*(3.34)	0.88(0.84–0.91)	0.68(0.65–0.71)	0.75(0.72–0.78)
>30	43,899*(3.32)	0.83(0.80–0.86)	0.60(0.58–0.62)	0.70(0.68–0.73)	43,864*(3.34)	0.92(0.89–0.95)	0.66(0.64–0.69)	0.76(0.74–0.79)
Other non-BZDs only	14,268*(1.08)	1.25(1.19–1.32)	0.90(0.86–0.95)	1.00(0.95–1.05)	14,123*(1.08)	1.28(1.21–1.35)	0.92(0.88–0.97)	1.01(0.96–1.07)
<7 (cDDD)	4,383*(0.33)	1.36(1.24–1.50)	0.99(0.90–1.09)	1.10(1.00–1.20)	4,340*(0.33)	1.38(1.26–1.53)	1.01(0.92–1.11)	1.11(1.01–1.23)
7–30	6,549*(0.50)	1.12(1.04–1.22)	0.82(0.76–0.89)	0.89(0.82–0.97)	6,496*(0.49)	1.15(1.06–1.25)	0.85(0.78–0.92)	0.91(0.84–0.99)
>30	3,336*(0.25)	1.34(1.23–1.47)	0.94(0.86–1.03)	1.06(0.97–1.16)	3,287*(0.25)	1.36(1.24–1.49)	0.95(0.87–1.05)	1.06(0.97–1.16)
BZDs only	91,209*(6.91)	2.22(2.18–2.26)	1.56(1.53–1.59)	1.81(1.78–1.85)	90,093*(6.86)	2.34(2.30–2.39)	1.64(1.61–1.68)	1.90(1.86–1.93)
<7 (cDDD)	47,437*(3.59)	2.42(2.36–2.47)	1.69(1.65–1.73)	1.94(1.90–1.99)	46,869*(3.57)	2.57(2.51–2.63)	1.79(1.74–1.83)	2.05(2.00–2.10)
7–30	22,343*(1.69)	1.98(1.92–2.05)	1.43(1.39–1.48)	1.66(1.60–1.72)	22,065*(1.68)	2.07(2.00–2.14)	1.49(1.44–1.54)	1.72(1.66–1.78)
>30	21,429*(1.62)	2.05(1.98–2.11)	1.43(1.39–1.48)	1.68(1.62–1.73)	21,159*(1.61)	2.16(2.09–2.23)	1.50(1.45–1.55)	1.74(1.68–1.80)
Mixed	152,695(11.56)	1.74(1.71–1.77)	1.08(1.06–1.10)	1.44(1.42–1.47)	152,345(11.60)	1.93(1.89–1.96)	1.18(1.16–1.20)	1.54(1.52–1.57)
<7 (cDDD)	4,655*(0.35)	2.73(2.58–2.89)	1.73(1.63–1.83)	2.19(2.07–2.32)	4,622*(0.35)	3.00(2.83–3.19)	1.88(1.77–1.99)	2.37(2.23–2.52)
7–30	23,677*(1.79)	2.03(1.97–2.10)	1.34(1.30–1.38)	1.69(1.63–1.74)	23,588*(1.80)	2.24(2.16–2.31)	1.46(1.41–1.51)	1.83(1.77–1.89)
>30	124,363*(9.42)	1.66(1.63–1.68)	1.02(1.00–1.04)	1.37(1.34–1.39)	124,135*(9.45)	1.84(1.80–1.87)	1.11(1.09–1.13)	1.46(1.43–1.49)

Mixed indicates those who used at least two types of hypnotics: zolpidem and other non-BZDs, or non-BZDs and BZDs.

BZDs = benzodiazepines; CCI = Charlson comorbidity index; HR = hazard ratio; CI = confidence interval; cDDD = cumulative defined daily dose.

^a^ HR has been adjusted for age and gender.

^b^ HR has been adjusted for age, gender, level of main medical facility, area of residence, and CCI.

^c^ HR has been adjusted for age, gender, level of main medical facility, area of residence, and Elixhauser comorbidities.

### Matching analysis results

Table 4 shows the association between the use of hypnotics and mortality using two matching methods. The model in which people were matched by gender and age did not exhibit any significant differences from the model that used PSM. The model matching for gender and age showed that zolpidem lowered the risk of mortality by 24–33% compared to the non-users, and PSM demonstrated that zolpidem lowered the risk of mortality by 35–37%.

**Table 4:** Crude and adjusted risk of mortality for age- andgender-matching and propensity score matching.

	Age- and gender-matched model							
	Death/ N	CHR(95% CI)		AHR^a^(95% CI)	AHR^b^(95% CI)		AHR^c^(95% CI)	
Non-user	28,959 / 366,080	1.00		1.00	1.00		1.00	
Zolpidem only	7,032 / 120,881	0.89(0.87–0.92)		0.90(0.88–0.93)	0.67(0.66–0.69)		0.76(0.74–0.79)	
Other non-BZDs only	1,358 / 13,676	1.56(1.48–1.65)		1.28(1.22–1.35)	0.92(0.87–0.97)		1.02(0.96–1.07)	
BZDs only	15,229 / 87,326	2.86(2.81–2.92)		2.31(2.26–2.35)	1.61(1.57–1.64)		1.86(1.82–1.90)	
Mixed	23,039 / 144,706	2.55(2.51–2.59)		1.80(1.77–1.83)	1.12(1.09–1.14)		1.48(1.45–1.51)	
	Propensity score matching model							
	Model 1				Model 2			
	Death/ N	CHR(95% CI)	AHR^a^(95% CI)	AHR^b^(95% CI)	Death/ N	CHR(95% CI)	AHR^a^(95% CI)	AHR^c^(95% CI)
Non-user	19,685 / 272,795	1.00	1.00	1.00	17,740 / 254,233	1.00	1.00	1.00
Zolpidem only	3,642 / 99,235	0.50(0.48–0.51)	0.58(0.56–0.61)	0.63(0.61–0.65)	3,590 / 95,507	0.53(0.51–0.55)	0.63(0.61–0.65)	0.65(0.62–0.67)
Other non-BZDs only	710 / 10,201	0.96(0.89–1.04)	0.97(0.90–1.04)	0.99(0.92–1.06)	680 / 9,782	1.00(0.92–1.08)	1.01(0.94–1.09)	0.95(0.88–1.03)
BZDs only	7,984 / 66,344	1.71(1.67–1.76)	1.69(1.65–1.74)	1.73(1.68–1.77)	7,803 / 63,545	1.82(1.77–1.86)	1.81(1.76–1.85)	1.75(1.71–1.80)
Mixed	10,008 / 97,015	1.43(1.40–1.47)	1.25(1.22–1.28)	1.18(1.15–1.21)	9,155 / 85,399	1.55(1.51–1.59)	1.33(1.30–1.37)	1.35(1.32–1.39)

Mixed indicates those who used at least two types of hypnotics: zolpidem and other non-BZDs, or non-BZDs and BZDs.

BZDs = benzodiazepines; CHR = crude hazard ratio; AHR = adjusted hazard ratio; CI = confidence interval.

Model 1 is propensity score matching for age, gender, level of main medical facility, area of residence, and CCI.

Model 2 is propensity score matching for age, gender, level of main medical facility, area of residence, and Elixhauser comorbidities.

^a^ HR has been adjusted for age and gender.

^b^ HR has been adjusted for age, gender, level of main medical facility, area of residence, cDDD, and CCI.

^c^ HR has been adjusted for age, gender, level of main medical facility, area of residence, cDDD, and Elixhauser comorbidities.

### Secondary analysis on zolpidem users

Table 5 shows the analytical results of specific causes of death. Cancer exhibited the highest risk of mortality, septicemia did not show any statistical significance, and all other specific causes of death indicated that the use of zolpidem involved a low risk of mortality.

**Table 5:** Analysis of the causes of death of the study subjects who only used zolpidem.

	Non-user		Zolpidem user			
	Number of deaths (%)	HR	Number of deaths (%)	HR^a^ (95% CI)	HR^b^ (95% CI)	HR^c^ (95% CI)
Cause-specific						
Cancer	5,019(9.66)	1.00	2,030(25.76)	2.27 (2.16–2.39)	1.11(1.05–1.17)	1.65(1.57–1.74)
Cardiovascular conditions	5,023(9.67)	1.00	994(12.62)	0.93 (0.87–1.00)	0.75(0.70–0.80)	0.76(0.71–0.82)
Respiratory conditions	10,871(20.93)	1.00	1,358(17.24)	0.67 (0.64–0.71)	0.55(0.52–0.58)	0.58(0.55–0.61)
Accident	4,627(8.91)	1.00	390(4.95)	0.61 (0.55–0.68)	0.55(0.50–0.61)	0.59(0.53–0.66)
Digestive disease	8,374(16.12)	1.00	751(9.53)	0.61 (0.57–0.66)	0.49(0.45–0.53)	0.53(0.50–0.58)
Other	18,020(34.70)	1.00	2,355(29.90)	0.77 (0.73–0.80)	0.62(0.59–0.65)	0.66(0.63–0.69)
Total	51,934(100.00)		7,878(100.00)			

HR = hazard ratio; CI = confidence interval.

^a^ HR has been adjusted for age, and gender.

^b^ HR has been adjusted for age, gender, level of main medical facility, area of residence, cDDD, and CCI.

^c^ HR has been adjusted for age, gender, level of main medical facility, area of residence, cDDD, and Elixhauser comorbidities.

Table 6 demonstrates the results of the secondary analysis regarding the duration of zolpidem use. Compared to the non-users or users with a lower dosage (Model 1), the results showed that the risk of mortality decreased with an increased duration of usage, regardless of whether comorbidities were adjusted for using the CCI or Elixhauser indices. The strongest protective effect was found in the subjects who had taken hypnotics for over a year, with a 43–54% lower risk of mortality. When the group with the lowest duration of use was set as the reference (Model 2), the group taking hypnotics for over a year still had a mortality reduction of 41–43%.

**Table 6:** Analysis of the dose-response relationship of mortality with the duration of zolpidem use.

		Model 1			Model 2		
Days	Death / N (%)	HR^a^ (95% CI)	HR^b^ (95% CI)	HR^c^ (95% CI)	HR^a^ (95% CI)	HR^b^ (95% CI)	HR^c^ (95% CI)
Non-user	51,934 /938,062(5.54)	1.00	1.00	1.00			
<30	4,698 /77,251(6.08)	0.88(0.85–0.91)	0.69(0.67–0.72)	0.78(0.76–0.81)	1.00	1.00	1.00
30–179	2,257 /31,652(7.13)	0.91(0.87–0.95)	0.70(0.67–0.73)	0.83(0.80–0.87)	1.00(0.96–1.05)	0.94(0.90–0.99)	0.99(0.94–1.04)
180–364	427 /6,729(6.35)	0.75(0.68–0.83)	0.55(0.50–0.61)	0.68(0.62–0.75)	0.82(0.75–0.91)	0.71(0.64–0.78)	0.76(0.69–0.84)
≧365	496 /8,454(5.87)	0.63(0.58–0.69)	0.46(0.42–0.51)	0.57(0.52–0.62)	0.70(0.64–0.77)	0.57(0.52–0.63)	0.59(0.54–0.65)

^a^ HR has been adjusted for age, and gender.

^b^ HR has been adjusted for age, gender, level of main medical facility, area of residence, and CCI.

^c^ HR has been adjusted for age, gender, level of main medical facility, area of residence, and Elixhauser comorbidities.

The non-user group was set as the reference group in Model 1, and the group with less than 30 days of use was set as the reference group in Model 2.

## Discussion

In this ten-year cohort study, we analyzed the relationship between hypnotic usage and mortality. Our results showed that the BZD group and the mixed hypnotics group had higher risks of mortality compared to the non-users, whereas the zolpidem group showed a lower risk of mortality. Analyses using different matching methods yielded similar results. The relationship between zolpidem and specific diseases with respect to mortality rates showed that among all the specific diseases analyzed only cancer exhibited a higher risk of mortality, whereas other diseases indicated lower risks of mortality. This suggests that the reduction of mortality risks for specific causes of death among zolpidem users was not restricted to a single disease. Furthermore, the risk of mortality decreased with increases in the cumulative zolpidem administration period, suggesting a significant dose-response relationship between zolpidem and mortality.

Our results are compatible with the findings of studies conducted in other countries, which showed that taking BZDs could lead to a higher risk of mortality [8, 9, 13, 17, 20]. However, the results of studies on zolpidem have not been consistent. In two of the above studies, one found no significant relationship between total death and zolpidem use [16], whereas the more recent study with a larger sample size found that the use of zolpidem was associated with higher risks of mortality and incident cancer [20]. Another study based on medical claims data, subjects taking zolpidem had a higher risk of cancer [27]. Although our results also indicated that the subjects taking zolpidem displayed higher risks of cancer-induced death, the overall risk of mortality was significantly lower. The possible reasons for the increased cancer risk may be the regurgitation that increases the risk of cancer of the upper digestive tract, or more health attention and surveillance including cancer detection for zolpidem users that facilitates the discovery of screening-related cancers [20, 27].

Zolpidem is a short-acting hypnotic that rapidly induces sleep and shortens sleep induction time [28]. It is an effective treatment for the insomnia characterized by an inability to fall asleep. Since the 1960s a number of studies have enriched our understanding of the effect of sleep on health [29–31]. Other studies have further confirmed the relationship between poorer sleep quality and an increased risk of mortality [10]. Research relevant to sleep duration, mortality, and chronic diseases has revealed the importance of sleep duration on the risk of mortality [32, 33] as well as chronic diseases such as cardiovascular diseases [34, 35] and diabetes [36]. Thus, a plausible explanation of our findings is that individuals who want to sleep better use zolpidem to shorten sleep induction time and improve sleep [37, 38]. As a result, their overall risk of mortality is then reduced as compared with the non-users, with some of whom may also have sleep problems and poor sleep quality. Since the sleep information was not available in our study, similar investigation with sleep-related information is still needed.

In addition, zolpidem, as previously indicated, is the most commonly prescribed hypnotics in Taiwan. Physicians are allowed to prescribe zolpidem instead of BZDs to patients with complaints related to sleep but not necessarily a definitive diagnosis of insomnia. It is therefore possible that zolpidem not only treats symptoms related to insomnia but also facilitates sleep and, consequently, provides better sleep quality. Thus, in this study, where we controlled for possible confounding factors, the zolpidem but not BZDs users had a lower risk of mortality compared to the non-users.

This study is unique for a number of reasons. First, the data are representative of the total population in Taiwan, with a large number of study subjects and a long follow-up period. Second, because zolpidem is the most commonly prescribed hypnotic in Taiwan, it was possible to determine the risk of mortality specifically for zolpidem alone. Third, several methods of adjustment for potential confounders or bias were employed, including the exclusion of subjects who died one year after the beginning of observation, and matching for potential confounders using PSM or gender- and age-matching. In addition, the results of models that separately adjusted for indices containing multiple diseases or individual diseases were similar (results not shown), indicating that our results after adjusting for diseases were robust. Finally, specific causes of death were analyzed for the patients using only zolpidem to identify the risk of mortality for each disease.

There are also several limitations in this study. First, the data contained in the NHIRD do not include certain factors potentially related to the use of hypnotics and mortality including lifestyle factors such as drinking, smoking, and physical activity, and genetic factors [39] and thus there may have been residual confounding effects. Second, it was not possible to determine whether the relationship between hypnotics and mortality was caused by medication or insomnia. Although numerous studies have indicated that deficiencies in sleep duration and quality increase the risk of death [10, 17, 32, 33], the independent effect of zolpidem on mortality reduction should be interpreted with caution. Third, the use of prescribed hypnotics in the claims data is inferred from prescription data and therefore may not accurately reflect the actual usage. This may have resulted in an overestimation of the use of hypnotics. However, even if the users did not take most of the prescribed hypnotics, our findings would underestimate the effect of the use of hypnotics on death rates. Fourth, some patients may have obtained hypnotics from sources other than NHI prescription and thus the actual figures regarding the use of hypnotics may have been underestimated. However, the effect of this underestimation on the results of this study was likely minimal, as all hypnotics in Taiwan are controlled drugs and require a prescription. Fifth, we assumed that the users took hypnotics on a long-term and regular basis. The duration of using hypnotics was calculated from the beginning of the study for all study subjects. Since the actual duration of using hypnotics was not considered, it is likely that the information bias exists in our results. However, the mortality risk may have been overestimated for those using before the beginning of the study and underestimated for those using after the beginning of the study. As a result, it is possible that this bias could be non-differential. Sixth, the severity of diseases, especially terminal illnesses, may be important confounding factor in the association between the use of hypnotics and death [40]. Unfortunately, disease severity was not collected in the NHIRD, and it was unable to establish whether the use of hypnotics was attributable to terminal illness. Therefore, our findings could be biased to some extent. If these factors were taken into consideration, the risk of mortality for users taking BZD or mixed users may have been reduced but the protective effect for zolpidem may have been stronger. Seventh, data on diseases and health care were collected from the baseline throughout the end of the study. It is possible that diseases and use of health care resulted from the use of hypnotics, and the adjustment for diseases and use of health care may have led to an over-adjustment which would have obscured the association. However, the results were similar even if data on diseases and the use of health care at baseline only were considered in the model. Eighth, a limitation of our study relying on the observational data is that the study cannot prove a causal relationship, but rather an association between hypnotics, especially zolpidem, and death. Further and larger studies, particularly randomized controlled trials, are needed to confirm whether it would be beneficial for people with sleeping problems to take zolpidem. Finally, the association of zolpidem with mortality was weak and should be interpreted with caution because it still leave room for some skepticisms, including possible bias and uncontrolled confounders mentioned above.

In summary, this investigation, which was a long-term observational study of a large sample found that the use of BZDs and combinations of multiple hypnotics was associated with an increased risk of mortality, whereas the use of zolpidem was associated with a lower risk of mortality. However, the mechanisms by which zolpidem reduces the risk of mortality, the roles of age and gender in the associations between hypnotics and mortality, and the factors affecting different specific causes of deaths (especially those with a significant risk reduction such as respiratory diseases, accidents, and digestive diseases), warrant further research.

## Supporting Information

S1 Text Data Availability Statement. (PDF)
