# Supplementary material for: The Use of Hypnotics and Mortality - A Population-Based Retrospective Cohort Study
Source: PLoS One. 2015 Dec 28;10(12):e0145271. doi: 10.1371/journal.pone.0145271 (PMC4692546; doi:10.1371/journal.pone.0145271)
Supplement: S1 Text — (PDF) [file pone.0145271.s001.pdf]

S1 Text. Data Availability Statement

All data and related metadata were deposited in an appropriate public repository. The data on the study population that were obtained from the National Health Insurance Research Database (NHIRD) (http://w3.nhri.org.tw/nhird//date_01.html) are maintained in the NHIRD (http://nhird.nhri.org.tw/). The National Health Research Institutes is a nonprofit organization established by the Taiwanese government. These data were released by the NHIRD for research uses. Every interested researcher is able to apply for and obtain the data in the same way as we did.
